# Isolation and functional diversification of dihydroflavonol 4-Reductase gene *HvDFR* from *Hosta ventricosa* indicate its role in driving anthocyanin accumulation

**DOI:** 10.1080/15592324.2021.2010389

**Published:** 2021-12-24

**Authors:** Shijie Qin, Yitong Liu, Baiqi Cui, Jianlin Cheng, Shuying Liu, Hongzhang Liu

**Affiliations:** College of Life Sciences, Jilin Agricultural University, Changchun, P.R. China

**Keywords:** Anthocyanins, dihydroflavonol 4-reduetase (DFR), *H. ventricosa*, ectopic expression

## Abstract

Anthocyanins are natural colorants are synthesized in a branch of the flavonoid pathway. Dihydroflavonol-4reductase (DFR) catalyzes dihydroflavonoids into anthocyanins biosynthesis, which is a key regulatory enzyme of anthocyanin biosynthesis in plants. *Hosta ventricosa* is an ornamental plant with elegant flowers and rich colorful leaves. How the function of HvDFR contributes to the anthocyanins biosynthesis is still unknown. In this study, the DFR homolog was identified from *H. ventricosa* and sequence analysis showed that HvDFR possessed the conserved NADPH binding and catalytic domains. A phylogenetic analysis showed that HvDFR was close to the clade formed with MaDFR and HoDFR in Asparagaceae. Gene expression analysis revealed that HvDFR was constitutive expressed in all tissues and expressed highly in flower as well as was positively correlated with anthocyanin content. In addition, the subcellular location of HvDFR showed that is in the nucleus and cell membrane. Overexpression of HvDFR in transgenic tobacco lines enhanced the anthocyanins accumulation along with the key genes upregulated, such as F3H, F3ʹH, ANS, and UFGT. Our results indicated a functional activity of the HvDFR, which provide an insight into the regulation of anthocyanins content in *H. ventricosa.*

## 1. Introduction

Anthocyanins are responsible for the appearance of colorful tissues in plants, like pink, purple, bule or red.^1^Anthocyanins are mainly distributed in the vacuoles of leaves, petals, and rhizomes, which participate in the defense process against the damage caused by biotic or abiotic stresses.^2,3^ For human, anthocyanins have been believed as health benefits diet due to their strong antioxidant properties.^4^ For ornamental plants, flower color is an important feature, which is related to their petal structures, types, and quantities of pigments within the petals, among which pigments play a pivotal role to decide flower color.^5,6^ Flavonoids, a class of main pigments, can produce a full spectrum of colors from pale yellow to blue violet. Among them anthocyanins are also the major components of flavonoids, showing a wide range of colors from pink to purple, and play an irreplaceable role in the formation of flower colors in plants.^7,8^

The biosynthetic pathways and key genes of anthocyanins in plants have been reported in detail, which consists of three stages.^1,9–12^ First, the conversion of phenylalanine to coumarin-CoA catalyzed by phenylalanine ammonia lyase (PAL), cinnamic acid-4-hydroxylase (C4H) and 4-coumaric acid-CoA ligase (4 CL). Second, one coumarate-CoA molecule and three malonyl-CoA molecules are catalyzed by chalcone synthase (CHS), chalcone isomerase (CHI), flavanone-3-hydroxylase (F3H), flavonoid 3′-hydroxylase (F3′H) and flavonoid 3′5′-hydroxylase (F3′5′H) to generate dihydroflavonol. Finally, dihydroflavonol 4-reductase (DFR) and anthocyanin synthase (ANS) as well as flavonoid glucosyltransferase (UFGT) and methyltransferase (MT) catalyze the synthesis of various types of anthocyanins by dihydroflavonol. Among them, DFR catalyzed the reaction of the dihydromyricetin to leucodelphinidin, which is the first committed reaction leading to anthocyanin production and a key enzyme spot regulating the carbon flux direction in the anthocyanin accumulation.^13,14^

Owing to the key role in the anthocyanin biosynthesis, DFR genes were isolated and functional characterized in many plants, including petunia, mulberry, wheat,^15–17^ and other ornamental plants, such as *Angelonia x angustifolia*,^18^ *Calibrachoa hybrida*,^19^ *Agapanthus praecox*,^20^ and crabapples and grapes.^21,22^ The functions of DFR genes have been shown to be related to anthocyanin synthesis. For example, the CRISPR/Cas9-mediated mutagenesis of *DFR* in *Ipomoea nil* displayed white flowers with a great loss of floral anthocyanins.^23^ Also, three genes *F3ʹH, DFR*, and *LDOX* were significantly related to seed color and anthocyanin content in black rice.^24^ In tobacco, RNAi-mediated silencing of *DFR* controlled the flower color from purple to white, which indicated the DFR turned the gene flux in flavonoid biosynthesis.^25^ On the other hands, overexpression *CsDFR* made early flowering and enhanced biotic stress tolerance in tobacco.^26^ Similarly, *TaDFR* could recovery the anthocyanin accumulation and tolerance to stress like UV-B, cold and high salt in *Arabidopsis dfr* mutant.^27^

*Hosta ventricosa*, a species in the family Liliaceae, is an important landscaping plant and herbaceous ornamental flower.^28^ *H. ventricosa* is cold-resistant and shade-loving, and mainly distributed in temperate and subtropical regions of Asia.^29,30^ The wild type *H. ventricosa* has only two flower colors, purple and white, which is a good material to study the mechanism of flower color formation. In our previous study, the competitive transcriptomes were generated between color different flowers of *H. ventricosa*, in which thousands DEGs were selected.^31^ However, the key genes involved in anthocyanins biosynthesis still need to be experimentally identified. Here, the DFR homolog from *H. ventricosa* was isolated and characterized, and its roles in the regulation of anthocyanins was also revealed. Our work provides a theoretical basis for the application of DFR in molecular color breeding in ornamental plants.

## 2. Materials and methods

### 2.1. Plant materials and growth conditions

*H. ventricosa* plantlets were collected from the Greenhouse A5 in Jilin Agricultural University Experimental Garden, in Jilin province, China. The tissues of roots, stems, leaves, and flowers in different flowering stages were quickly frozen with liquid nitrogen and stored at −80 ◦C for later use. *N. tabacum“NC89”*was planted for the transformation. The seedlings were cultured in a growth chamber with growing condition: temperature, 25 ◦C, humidity, 70%, illumination, 6000 Lx, and light cycle: 16/8 h.

### 2.2. Cloning and bioinformation analysis of HvDFR

Total RNA was extracted using RNAprep Pure Extraction Kit (DP441, TIANGEN biochemical Technology, Beijing, China) and checked the quality and integrity by the 1.0% agarose gel electrophoresis analysis. And the first strand of cDNA was synthesized by PrimeScript RT reagent Kit with gDNA (RR047, TaKaRa, Tokyo, Japan) according to the instructions. The specific primers were designed using Permier Primer 6.0 based on the sequences from the transcriptome data (Table S1). The HvDFR gene was amplified from the cDNA with primers using TaKaRa Taq™ (R001A, TaKaRa, Tokyo, Japan), with the following cycle conditions: initial denaturation at 94◦C for 3 min, dolllowed by 33 cycles of denaturation at 94◦C for 30s, annealing at 58◦C for 30s, and extension at 72◦C for 30s, and a final extension at 72◦C for 10 min. The PCR fragments were obtained and purified with Gel Extraction Kit (CW2302 CWBIO,JiangSu,China), and then cloned into a pMD™ 18-T Vector Cloning Kit (6011 Takara, Tokyo, Japan) and sequenced by GENE Biotechnology company (Beijing, China). The conserved domain of the candidate gene was predicted on the NCBI Conserved Domain Database (https://www.ncbi.nlm.nih.gov/structure/cdd/wepsb.cgi). The alignment analysis of DFRs were employed with DNAMAN and a phylogenetic tree was constructed with MEGA 7.0 using the Nerghbor-Joining method and 1000 bootstrap replicates.

### 2.3. Quantitative PCR analysis

The first strand of cDNA for quantitative PCR was synthesized using PrimeScript RT reagent Kit with gDNA Eraser (RR047, TaKaRa, Japan). The reaction was performed on BioRad IQ5 (Biorad, USA) with the SYBR Prime Ex Tap II (RR420, TaKaRa, Japan) according to the user’s manual. The reaction procedure was as follows: pre-denaturation at 95 ◦C for 30 s; 98 ◦C 5 s, 60 ◦C 34 s, 40 cycles; 95 ◦C 15 s, 60 ◦C 1 min, 95 ◦C 15 s. The relative expression level of *HvDFR* in different tissues and different development stages of flower was measured with *HvActin* as the housekeeping.^31^

The key genes involved in anthocyanin biosynthesis in tobacco were also determined by quantitative PCR. The reaction system and procedures were as same as above, and the primers were listed in Table S1. And the relative expression levels of the key genes were compared with the reference gene in control with the 2 ^−∆∆CT^ method.^32^ The results are presented as the means ± standard errors, and the significance analyses of gene expression levels were performed using Student's t-test (*p* <.05).^33^

### 2.4 Construction of overexpression vector and Agrobacterium-mediated transformation

The PCR products were digested by *Bstp* I and *Bgl* II and then cloned into a pCAMBIA3301 vector using the same restriction sites. Then the recombined vector was transformed to the *Agrobacterium* GV3101 strain by the thermal shock method,^34^ and the specific PCR was preformed to verify the transformation. The process of tobacco transformation was performed using the leaf plate method as previously described.^35^ The independent transgenic lines with the highest *HvDFR* gene expression level were selected for further experiments by qRT-PCR confirmation.

### 2.5 The subcellular localization of HvDFR

The recombined vector HvDFR-GFP was transformed to the Agrobacterium GV3101 and set the empty vector as control. To perform tobacco infiltration analysis, the transformed Agrobacterium GV3101 was suspended using induction medium (10 mM/L MES + 10 mM/L MgCl_2_ + 100 uM/L AS) and injected into the *N. benthamiana NC89* leaf.^36^ Then, the GFP signals were detected 2 ~ 5 days after injection by a Leica SP8 Meta device (Leica, Germany).

### 2.6. Total flavonoids and anthocyanin determination

Total flavonoids were estimated according to the aluminum chloride method.^37^ Briefly the flash tobacco flowers from transgenic and wildtype lines were collected and ground to a fine powder with liquid nitrogen. The powders were extracted with 10 mL of 95% methanol under sonication for 5 h at 60°Cand then centrifuged at 10,000 g for 15 min. The supernatant was transferred to add 1 ml 10% AlCl3, and constant volume to 10 ml. The absorbance for each sample was measured at 420 nm. Rutin concentrations ranging from 0 to 9 μg/mL were prepared, and the standard calibration curve was obtained using a linear fit (*R^2^* = 0.9995). Samples from flowers were extracted for anthocyanins measurement, and quantification of anthocyanins was performed according to the protocols of Ni et al.^38^ The samples were analyzed in triple.

## 3. Results

### 3.1. Molecular characterization of HvDFR reveals its conserved sequence structure

**Figure 1.**
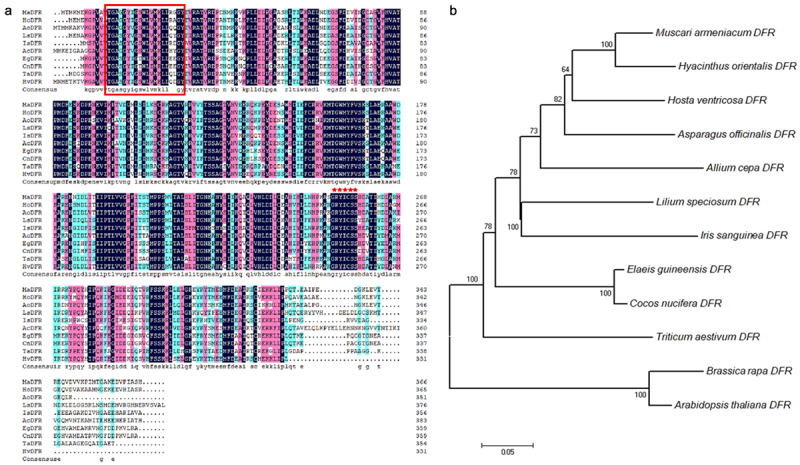
Amino acid alignment and phylogenetic analysis of DFRs from plants with HvDFR. (a) Alignment of amino acid sequences encoded by 9 DFRs genes. MaDFR, *Muscari armeniacum* DFR, HoDFR, *Hyacinthus orientalis* DFR, AoDFR, *Asparagus officinalis* DFR, AcDFR, *Allium cepa* DFR, LsDFR, *Lilium speciosum* DFR, IsDFR, *Iris sanguinea* DFR, EgDFR, *Elaeis guineensis* DFR, CnDFR, *Cocos nucifera* DFR, TaDFR, *Triticum aestivum* DFR. The black, pink, and bule color means 100%, >75%, and >50% homology among those sequences, respectively. The red boxed region is conserved NADPH-binding sites, the red star labeled sites indicated the substrate specificity. (b) Phylogenetic tree of HvDFR constructed with MEGA 7.0, using the Neighbor-joining (NJ) method and 1000 bootstrap replicates.

To further study the gene function of *HvDFR*, we designed gene-specific primers (Table S1) based on the contig sequences from our former transcriptome database^31^ and obtained the full-length transcript sequence of HvDFR. The CDS of HvDFR encoded a protein of 332 amino acids, contained a conserved NADPH binding domain from V14 to Y34 (VTGASGYIGSWLVMKLLQDGY) motif (Figure 1a). Also, we performed multi-sequences alignment analysis of HvDFR with DFRs in other plant species. And it was found that all sequences aligned contained the conserved NADPH binding motif at the N-terminal, while that at the C-terminal was quite various (Figure 1a). These DFR proteins all contained NADPH coenzyme binding site, active site, and substrate binding site. The amino acid sequence of NADPH binding site and active site are relatively conserved, while the amino acid sequence of the substrate binding site varies greatly. It is showed that DFR has specific catalytic roles in plants.

In addition, a phylogenetic tree was generated to investigate the homology of HvDFR to other DFRs from *H. ventricosa* and 11 other plants. The amino sequences were used to analyze and showed that HvDFR was close to the clade formed with MaDFR and HoDFR in Asparagaceae (Figure 1b). The highly conserved homology within the family indicated the importance of DFR function.

### 3.2. *Expression pattern of* HvDFR *is positively correlated with anthocyanin content*

**Figure 2.**
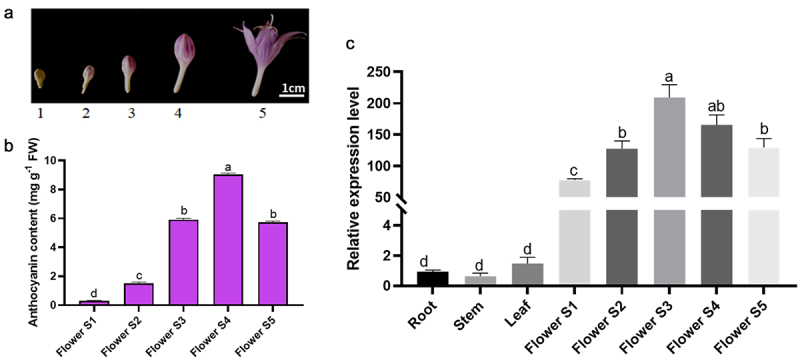
Anthocyanin content and relative gene expression level of *HvDFR* in different tissues and flowering stages in *H. ventricosa*. Note, a, b, c, and d represent significant differences between samples.

Firstly, we detected the anthocyanin content in flowering stages (Figure 2a,b) to figure out the accumulation profile in the petals in 5 different stages. The results showed that the anthocyanin level was variational with flowering time and the highest anthocyanin content was 9.04 mg·g^−1^ in the Flower S4 stage. Furthermore, to study the expression profiles of HvDFR gene, the quantitative PCR analysis of HvDFR in tissues was preformed (Figure 2c). The HvDFR was expressed in root, stem, leaf, and flower tissues, and it maintained a high level in flower. Therefore, we also detected the HvDFR expression in flowering stages. It showed that the expression level of HvDFR was increased with flowering stages and reached the highest expression in the Flower S3 when the flower is still the bud not open.

Besides, it is positively correlated with the anthocyanin content and HvDFR expression level (Figure 2b,c). The results indicated that a high level of *HvDFR* expression enhanced anthocyanin biosynthesis and potentially caused the darker purple color in flowers of *H. ventricosa*.

### 3.3. Subcellular localization of HvDFR was in the nucleus and cell membrane

**Figure 3.**
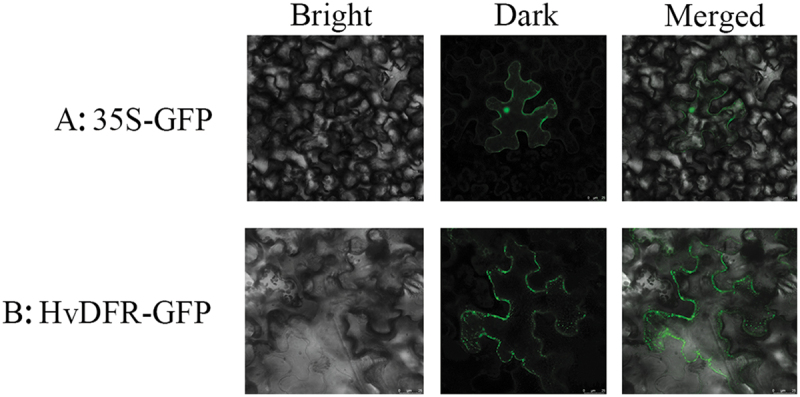
The subcellular localization of HvDFR. (a) observation of GFP empty vector introduced in tobacco leaf. (b) Observation of HvDFR-GFP recombinant vector introduced in tobacco leaf.

To investigate the subcellular localization of HvDFR, the HvDFR coding sequence was cloned and fused to the eGFP reporter under the CaMV35S promoter and then was transient to tobacco leaves. Figure 3(a) showed that the free eGFP signals were appeared in the nucleus, cytoplasm, and cell membrane, while the HvDFR-GFP fluorescence was observed in the nucleus and the cell membrane (Figure 3b).

### 3.4. Overexpression of HvDFR transgenic tobacco lines were generated by Agrobacterium-mediated transformation

**Figure 4.**
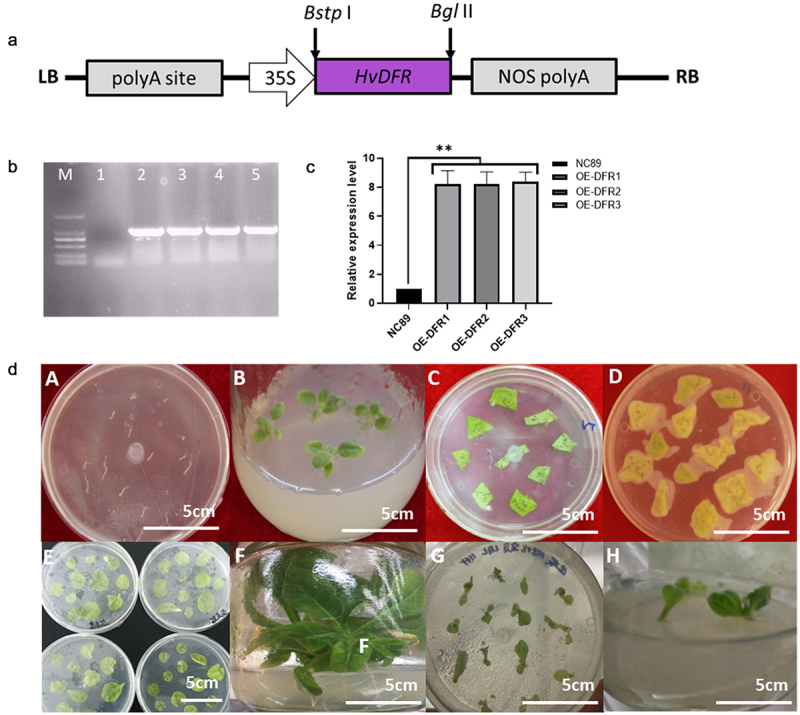
Construction of the overexpression vector and genetic transformation procedure. (a) The schematic of the *HvDFR* gene overexpression cassette. (b) Agarose gel showing the specific fragment for the *HvDFR* in the transgenic tobacco lines, M, DL 2000 DNA marker, 1–5, WT control and transgenic tobacco lines 1–4. (c) the relative expression level of *HvDFR* in the transgenic tobacco lines. (d) The process of *Agrobacterium*-mediated transformation in tobacco.

The transgenic tobacco lines overexpressing *HvDFR* under the control of 35S promoter were developed through *Agrobacterium tumefaciens*-mediated transformation. The schematic representation of the T-DNA region of pCAMBIA3301-HvDFR is shown in Figure 4(a). The pCAMBIA3301-HvDFR plasmid was transferred into *A. tumefaciens EHA105* by the freeze-thaw method and verified with *HvDFR* specific PCR analysis (Figure 4b). The regenerated plantlets were screened on MS plates-containing hygromycin (50 mg/ml). The resistant shoots were regenerated from the cut surface of the explants and these shoots were separated from the mother explants and roots induced. The introduction of *HvDFR* gene was confirmed by gDNA PCR from leaves of transgenic plants with gene-specific primers. A specific band of 900 bp indicated the introduction of *HvDFR* in transgenic tobacco (Figure 4c). The plants transformed with an empty vector pCAMBIA3301were served as control. We isolated 15 individual transgenic plants following *A. tumefaciens*-mediated genetic transformation (Figure 4d). We selected overexpression tobacco lines OE-DFR1, OE-DFR2 and OE-DFR3 for further examination due to *HvDFR* highest expressed, while no visible phenotypes were observed in the transgenic lines.

### 3.5. *Total flavonoids and anthocyanin concentrations were increased by overexpression of* HvDFR *in tobacco*

**Figure 5.**
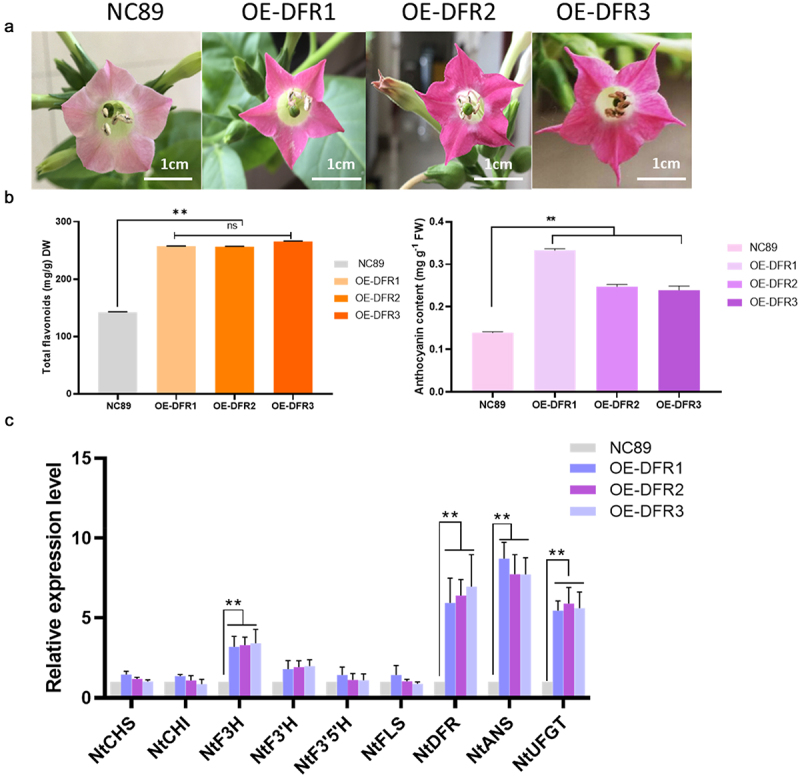
The phenotype and flavonoids, anthocyanins and related genes expression level in OE tobacco lines compared with the control. (a) The 3 days flowers of transgenic tobacco and the wildtype. (b) Total flavonoids and anthocyanin contents in OE lines and NC89. (c) The key genes involved in anthocyanin biosynthesis in flowers of transgenic lines compared with the control. Asterisks, significant differences, *p* < .01; ns, not significant, *p* > .05; student’s t-test.

DFR plays a key role in anthocyanin biosynthesis in plant. To study the functions of *HvDFR* we generated the overexpression tobacco lines and measured the total flavonoids and anthocyanins content in our transgenic tobacco lines OE-DFR1, OE-DFR2 and OE-DFR3. In the results, the flavonoids concentrations in wildtype NC89 and OE lines were 142.39, 257.20, 256.38, 265.61 μg/g, respectively, which observed significantly increased in overexpression of *HvDFR* lines. In addition, the results showed that the anthocyanin contents in OE tobacco lines were 0.333, 0.248, 0.239 mg/g which were 2.40 -fold, 1.78 -fold, and 1.72 -fold, respectively, compared with the wildtype NC89 (Figure 5a,b).

Meanwhile, we determined the key genes involved in anthocyanin biosynthesis by qRT-PCR in tobacco overexpression lines. As expected, the relative expression of *HvDFR* in OE lines had exceeded 8 folds than the control (Figure 5c). It also showed that the expression levels of *F3H, F3ʹH, DFR, ANS, UFGT* were significantly increased in overexpression tobacco lines compared to the control. While the *CHS, CHI, F3ʹ5’H*, and *FLS* were no significance in non-/transgenic lines (Figure 5c).

## 4. Discussion

Anthocyanins biosynthesis in plant is derived from the branch of flavonoid synthesis. DFR gene are a key player in the production of anthocyanins and flavonoids, whose functional absence will result in the lack of anthocyanin accumulation thereby affecting flower color. Since the DFR gene was cloned in maize for the first time and successfully applied in genetic engineering to change the flower color of petunia, the DFR genes of many plants have been cloned and further applied to the transformation of flower color or fruit color through transgenic technology. In this study, we showed that amino acid sequence of HvDFR contained the conserved NADPH binding domain and catalytic site while the varies substrate binding site aligned with DFR proteins, indicating the DFR enzymes were important but specific diversity in plants. The sequences of DFR from the genus Asparagaceae were highly similar and the phylogenetic analysis also demonstrated that HvDFR was closely related to the clade of *Muscari armeniacum* and *Hyacinthus orientalis* (Figure 1), which t is consistent with that obtained using traditional taxonomy methods.

Previous studies have reported the expression patterns of DFRs, pointing out it expressed specifically in various organs which is related to its function. For example, GbDFRs were expressed in all tissues including root, stem, leaves and fruit with the highest expression in fruit.^38^ Also, 9 anthocyanin biosyntheitc genes such as *DFR, CHS, CHI, ANS* were expressed highly during fruit ripen in mulberry.^17^ Our results indicated that in Hv, HvDFR is broadly expressed in tissues incorporating roots, stems, leaves, and flowers, and HvDFR has the highest expression level in flowers. Further detailed analysis during five floral development stages has given out HvDFR expression is positively correlated with anthocyanins accumulation (Figure 2). This result could suggest HvDFR is a potential key candidate gene for anthocyanins biosynthesis in *H. ventricosa*. Therefore, our study further characterized the roles of HvDFR in regulating anthocyanins biosynthesis and changing floral color in *H. ventricosa*.

We further found that HvDFR localized in the nucleus and cell membrane through construction the recombine vector with GFP signal and transient transgenic system, which is consist with that of CnDFR in *Camellia nitidissima*. However, the subcellular location of DFRs is diverse in plants. For example, in ornamental kale (*Brassica oleracea* L. var. *acephala*) and grape hyacinth (*Muscari armeniacum*), the BoDFR and *GhDFR* genes locates in the cytoplasm and in the whole cells, respectively.^39,40^ Besides, protein–protein interaction assays revealed that the *AmCHI* and *AmDFR* were coexpressed in endoplasmic reticulum in *Antirrhinum majus*.^41^ Those results indicate that DFRs are functionally differentiated in various plants that is uniform with the variation in the amino acid sequence of DFRs.

It is a feasible and universal way to transform the target gene into *Arabidopsis* or *N. tabacum* who processing the stable transformation system to explore the gene function. In this study, the transgenic tobacco lines with overexpression of *HvDFR* were generated to further characterized the roles of *HvDFR* in regulating anthocyanins biosynthesis. It is found that there are darker purple in transgenic tobacco flowers accompanied with the anthocyanins and flavonoids accumulation (Figure 5b,c). Similarity, overexpression of *ApDFR* of *Petunia hybrida* with white flower resulted in a change in floral color to elegant lilac.^20^ Conversely, silencing of DFR will cause loss of plant anthocyanins and the resistance of abiotic stress resistance.^23^ All these research suggested that DFR is obviously enhanced the formation of anthocyanin in plants. Although we found that overexpression of *HvDFR* in tobacco can increase the levels of total flavonoids and anthocyanins, we should still detect the protein expression level and enzyme activity of *HvDFR* in transgenic tobacco lines to explore the deep understanding of catalytic function and the transcriptional regulation mechanism of HvDFR. Future studies determining of HvDFR catalytic activity *in vivo* or *in vitro* might be necessary to investigate the enzymatic characteristics of HvDFR.

The synthesis, transport and accumulation of anthocyanins are coordinately regulated by a variety of specific transcription factors. At present, the three most studied transcription factors in the anthocyanin synthesis pathway are MYB, bHLH and WD40 transcription factor families, which can regulate anthocyanins accumulation independently or in a complex. As a key enzyme in the anthocyanin synthesis pathway, DFR is under the transcriptional regulation of MYB transcription factor. For instance, MrMYB1 could upregulate anthocyanin biosynthetic pathway genes including *CHS, DFR, ANS* as well as *TT8* in MrMYB1-overexpressing *Arabidopsis*.^42^ A R2R3-MYB transcription factor MYB10 positively regulated the promoters of DFR resulted in the increasing of flavonoid, anthocyanins, and proanthocyanidins in *Prunus persica*.^43^ Therefore, cloning DFR can not only reveal the important role of DFR enzymes in different plant species on anthocyanin synthesis, but also serve as a regulatory site for the anthocyanin synthesis pathway, which is of significance for elucidating the transcriptional regulation of the anthocyanin pathway.

In the herbaceous peony, *Brunfelsia Acuminata* and *Phalaenopsis*,^44–46^ the anthocyanin synthesis pathways have been thoroughly studied, which have provided theoretical basis for the molecular breeding and germplasm innovation of those ornamental plants. However, in *H. ventricosa* the research is relatively lagging for the mining of gene/genome information, and color decision genes and their regulatory mechanisms are yet to be studied. Therefore, as the key gene for anthocyanin synthesis, the isolated and characterized HvDFR in this study could increase the content of anthocyanins and flavonoids in overexpression tobacco lines. It is a potential entry point for the germplasm improvement of *H. ventricosa* and provides a new insight for germplasm innovation and further breeding.

## 5. Conclusions

We identified the DFR homolog from *H. ventricosa*, and the sequence analysis showed that HvDFR possessed the conserved NADPH binding and catalytic domains. A phylogenetic analysis showed that HvDFR was close to the clade formed with MaDFR and HoDFR in Asparagaceae. Gene expression analysis revealed that HvDFR was constitutive expressed in all tissues and expressed highly in flower as well as was positively correlated with anthocyanin content. In addition, the subcellular location of HvDFR showed that is in the nucleus and cell membrane. Overexpression of HvDFR in transgenic tobacco lines enhanced the flavonoids and anthocyanins accumulation along with the key genes upregulated, such as *F3H, F3ʹH, ANS*, and *UFGT*. Our results indicated a functional activity of the HvDFR, which provide a new insight into the regulation of anthocyanins content as well as for germplasm innovation and further breeding in *H. ventricose.*

## Supplementary Material

Supplemental Material

## Abbreviations

4-coumaric acid-CoA ligase, 4CL; anthocyanin synthase, ANS; cinnamic acid-4-hydroxylase, C4H; chalcone isomerase, CHI; chalcone synthase, CHS; dihydroflavonol 4-reductase, DFR; flavanone-3-hydroxylase, F3H; flavonoid 3′-hydroxylase, F3′H; flavonoid 3′5′-hydroxylase, F3′5′H; flavonoid glucosyltransferase, UFGT; methyltransferase, MT; phenylalanine ammonia lyase, PAL.
