# Supplementary material for: Isolation and functional diversification of dihydroflavonol 4-Reductase gene HvDFR from Hosta ventricosa indicate its role in driving anthocyanin accumulation
Source: Plant Signal Behav. 2021 Dec 24;17(1):2010389. doi: 10.1080/15592324.2021.2010389 (PMC8967398; doi:10.1080/15592324.2021.2010389)
Supplement: Supplemental Material [file KPSB_A_2010389_SM5660.zip › Figure S.docx]

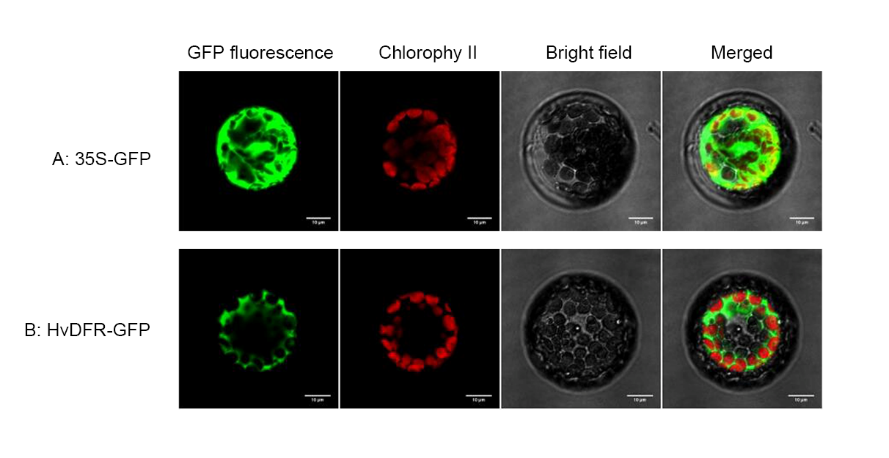
Figure S1 The subcellular localization of HvCHI by protoplast transformation. A, observation of GFP empty vector introduced in *Arabidoposis* protoplast. B, observation of HvDFR-GFP recombinant vector introduced in *Arabidoposis* protoplast.


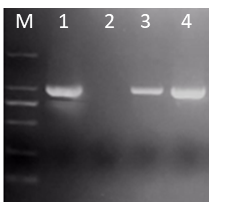


Figure S2 The agarose gel showing the specific fragment for the HvDFR in the Agrobacterium tumefaciens harbored the recombinant vectors.
