# Supplementary material for: Isolation and functional diversification of dihydroflavonol 4-Reductase gene HvDFR from Hosta ventricosa indicate its role in driving anthocyanin accumulation
Source: Plant Signal Behav. 2021 Dec 24;17(1):2010389. doi: 10.1080/15592324.2021.2010389 (PMC8967398; doi:10.1080/15592324.2021.2010389)
Supplement: Supplemental Material [file KPSB_A_2010389_SM5660.zip › Table S1 The primers used in this study.docx]

Table S1 The primers used in this study

| **Gene name** | **Sequences (5’-3’)** |
| --- | --- |
| DFR-F | ATGAATATGGAGACAAAAACGGTGAAG |
| DFR-R | TTAGAGAGGTATGAGCTTCTTCTCCATG |
| DFR-qPCR-F | AGCCAACAATCGACGGAGTG |
| DFR-qPCR-R | TCTCGTTGTACTCCGGCTTC |
| DFR-GFP-F | cggtacccggGGATCCATGAATATGGAGACAAAAACGGTGA |
| DFR-GFP-R | tgctcaccatGTCGACGAGAGGTATGAGCTTCTTCTCCATG |
| DFR-3301-F | AACCACCTTAATCTTCATGCTGCT |
| DFR-3301-R | AAGATTCAGATGCCCAGAGGTCCT |
| Q-HvActin-F | TCAAGCTGTGTTGTCCCTATAC |
| Q-HvActin -R | GGAAGGGCGTAACCTTCATAA |
| Q-NtActin-F | CGGCACGGATCGGTATATGAAGG |
| Q-NtActin-R | CAGGTACTGAGAACGGCAGCTTC |
| Q-NtCHS-F | AGTGGTGGTTGAAGTGCC |
| Q-NtCHS-R | CGCTTGACCGATGGAC |
| Q-NtCHI-F | CGGGTGCCTCCATTCTTT |
| Q-NtCHI-R  Q-NtF3H-F  Q-NtF3H-R  Q-NtF3‘H-F  Q-NtF3‘H-R  Q-NtF3’5’H-F  Q-NtF3’5’H-R  Q-NtFLS-F  Q-NtFLS-R  Q-NtDFR-F  Q-NtDFR-R  Q-NtANS-F  Q-NtANS-R  Q-NtUFGT-F  Q-NtUFGT-R | TTCGGCGATACTACACTTTGC  ATGCCCAACTTATCTCAC  TGGACCACTTCACCCT  GCACCCTACGGACCAAG  TGCGTCAGCAAACACCC  CTACCATACTTAGGAGCCA  TTTAGCAGCATCAGGAG  ATGGCTGTTGCTTCTACTC  CTTCTCGGCTCATTTCG  AACCAACAGTCAGGGGAATG  TTGGACATCGACAGTTCCAG  TGGCGTTGAAGCTCATACTG  GGAATTAGGCACACACTTTGC  GAGTGCATTGGATGCCTTTT  CCAGCTCCATTAGGTCCTTG |
